# Risk factors for absenteeism due to musculoskeletal diseases in workers in the judiciary sector

**DOI:** 10.47626/1679-4435-2021-634

**Published:** 2021-12-30

**Authors:** Rafael dos Reis França, Rita de Cássia Pereira Fernandes, Verônica Maria Cadena Lima

**Affiliations:** 1 Faculdade de Medicina da Bahia, Universidade de Federal da Bahia (UFBA), Salvador, BA, Brazil.; 2 Departamento de Estatística, Instituto de Matemática, UFBA, Salvador, BA, Brazil.

**Keywords:** absenteeism, sick leave, musculoskeletal diseases, occupational health, judiciary, absenteísmo, licença médica, doenças musculoesqueléticas, saúde do trabalhador, poder judiciário

## Abstract

**Introduction:**

Musculoskeletal diseases represent an important health problem for workers, due to the degree of suffering caused by pain and to the high frequency of absenteeism.

**Objectives:**

To identify risk factors for absenteeism due to musculoskeletal disease in employees at a judiciary court and to describe incidence, frequency, and duration of sick leaves.

**Methods:**

This is a 6-year follow-up study conducted in the state of Bahia, Brazil. The dependent variable was absenteeism, measured by the time of the first sick leave in the period. The Kaplan-Meier method was used to estimate survival functions, whereas risk factors for absenteeism were obtained by Cox regression.

**Results:**

Overall, 594 workers took sick leaves, with an incidence of 23% at the end of the period. The most frequent diagnoses were back pain (38.5%), shoulder lesions (11.7%), and synovitis and tenosynovitis (8.8%). Cases of one episode of sick leave per worker over the 6 years predominated (42.8%). Cox regression multivariate analysis identified the following variables as posing the greatest risk for sick leave: female sex (hazard ratio 1.39), age older than 40 years (hazard ratio 2.57), judicial technician workers (hazard ratio 1.48), and administrative workers (hazard ratio 1.30).

**Conclusions:**

Women, older adults, and individuals who hold technical positions are worthy of attention from health management department of the court, since they presented the highest rates of incapacity to work during the study period. Back pain was the main reason for musculoskeletal disability.

## Resumo

**Introdução:**

As doenças musculoesqueléticas representam um importante agravo na saúde dos trabalhadores devido ao grau de sofrimento gerado pelos quadros de dor e às elevadas frequências de absenteísmo.

**Objetivos:**

Identificar os fatores de risco para o absenteísmo por doença musculoesquelética em trabalhadores de um tribunal do setor judiciário e descrever a incidência, os indicadores de frequência e a duração das licenças médicas.

**Métodos:**

Estudo com seguimento de 6 anos, conduzido na Bahia. A variável dependente foi absenteísmo, medida através do tempo até a primeira licença médica no período. Utilizou-se o método de Kaplan-Meier para estimar as funções de sobrevida, enquanto os fatores de risco para o absenteísmo foram obtidos pela regressão de Cox.

**Resultados:**

Um total de 594 trabalhadores apresentaram licenças médicas, sendo a incidência ao final do período igual a 23%. Observou-se maior frequência de diagnósticos de dorsalgia (38,5%), lesões no ombro (11,7%) e sinovite e tenossinovite (8,8%). Predominaram casos de episódio único de licença médica por trabalhador ao longo dos 6 anos (42,8%). A análise múltipla com regressão de Cox identificou as seguintes variáveis como de maior risco para o afastamento do trabalho: sexo feminino (*hazard ratio* 1,39), idade acima de 40 anos (*hazard ratio* 2,57), cargo de técnico judiciário (*hazard ratio* 1,48) e atuantes na área administrativa (*hazard ratio* 1,30).

**Conclusões:**

Mulheres, pessoas com maioridade e indivíduos que ocupam cargo técnico devem receber atenção da gestão do tribunal, visto que apresentaram a maior incapacidade para o trabalho no período estudado. As dorsalgias figuraram como o principal motivo da incapacidade musculoesquelética.

## INTRODUCTION

Musculoskeletal diseases (MSD) are a group of diseases of the musculoskeletal system with varied clinical presentation and multifactorial and complex etiology. They represent an import health problem for workers, due to the high degree of suffering caused by pain and to the high frequency of absenteeism, in addition to incurring costs to the health care system and to the social security system.^1-3^

Among the cases of disability leave experienced by service and industry workers, the literature points to a great proportion of episodes of absenteeism due to musculoskeletal system diseases, followed by mental and behavioral disorders, injuries, poisoning, and some other consequences of external causes.^3,4^

The risk factors for sick leave due to MSD involve individual, sociodemographic, and organizational aspects.^5^ In the judiciary sector, workers develop complex and diversified activities, ranging from administrative tasks to procedural trials. These activities involve physical and psychological demands, which may occasionally contribute to the occurrence of MSD and disability due to these diseases.

Absenteeism due to MSD has a relevant economic impact. The number of records of sick leave among workers covered by the General Social Security System increased from 5,025 in 1988 to 30,334 in 2005, representing payments with benefits and compensations.^6^ In Brazil, costs with absenteeism, presenteeism, and early disability retirement are expected to account for 8.7% of the gross domestic product in 2030.^6^

Although absenteeism stands out as a relevant public health problem, with the literature showing several studies on this phenomenon in different working populations, few studies assessed absenteeism among workers in the judiciary sector. In this context, the present study aims 1) to identify the incidence of absenteeism due to MSD according to some epidemiological characteristics; 2) to describe indicators of duration and frequency of absenteeism according to diagnoses; and 3) to identify risk factors for absenteeism due to MSD.

## METHODS

This is a longitudinal study with 6-year follow-up (2011 to 2016) that assessed civil servants at a judiciary court in the state of Bahia, Brazil. Participants’ data were obtained from the Secretariat of Information and Communication Technology and from the Health Coordination. In the aforementioned court, judiciary (main activities) and administrative activities are performed in different sectors: labor courts, chambers, support centers, control stations, departments, and secretariats. The sectors are distributed in municipalities of the Salvador Metropolitan Region (four municipalities) and in the inland of the state of Bahia (28 municipalities). All civil servants who were active (with no sick leave due to MSD) on the date of study baseline (January 1, 2011) were eligible.

Absenteeism was defined according to the International Classification of Diseases - 10^th^ edition (ICD-10) code recorded in the certificates verified by an occupational physician of the institution’s health coordination. According to Ordinance no. 1,339/GM of the Brazilian Ministry of Health,^7^ sick leaves whose diagnoses were classified into following codes were included: G13, G47.2, G54.0, G54.1, G55.1, G56, G56.0, G56.1, G56.2, G56.3, G56.8, G56.9, G57.6, G61.8, G62.9, I73.0, I73.8, M058, M070, M13, M13.9, M19, M19.9, M22.4, M23, M23.3, M23.4, M23.5, M23.8, M23.9, M24.2, M24.5, M24.9, M25, M25.4, M25.5, M40.0, M43.1, M43.6, M46.1, M50, M50.0, M50.1, M50.2, M50.3, M50.8, M50.9, M51, M51.0, M51.1, M51.2, M51.3, M51.8, M51.9, M53, M53.1, M53.2, M53.3, M53.9, M54, M54.0, M54.1, M54.2, M54.3, M54.4, M54.5, M54.6, M60.9, M62, M62.6, M62.8, M63.8, M65, M65.2, M65.3, M65.4, M65.8, M65.9, M66, M66.2, M66.3, M66.4, M66.5, M67, M67.8, M67.9, M70, M70.0, M70.1, M70.2, M70.3, M70.4, M70.5, M70.8, M70.9, M71.9, M72.0, M72.2, M72.3, M75, M75.0, M75.1, M75.2, M75.3, M75.4, M75.5, M75.8, M75.9, M77, M77.0, M77.1, M77.4, M77.9, M79.1, M 79.2, M79.6, M79.8, M79.9, M87, M87.1, M87.3, M93.1, M93.8, M95.8, Z56.3, Z57.8, and Z57.9. According to this Ordinance,^7^ in addition to group M (work-related diseases of the musculoskeletal system and connective tissue), diseases in group G (work-related diseases of the nervous system) and group Z (stressful work schedule and occupational exposure to other risk factors) were included, as listed above.

On the date of study baseline (January 1, 2011), there were 2,674 active workers. Of these, six were excluded because they were absent due to MSD and six were excluded due to inconsistencies in information records. Therefore, the study included a total of 2,662 workers. Since it was a fixed cohort, new inclusions were not allowed after the date of study baseline.

Information for the organization of the database of the present study was obtained from the institution’s databases; sociodemographic and occupational variables were included. In addition to the number of episodes of sick leave due to MSD, the following variables were obtained for each episode: beginning date of sick leave, number of absent days, and diagnosis according to the ICD-10.

The description stage was succeeded by database organization. Annual cumulative incidences (number of new cases of sick leave/population exposed to the risk × 100) were calculated, described according to sociodemographic and occupational variables. This calculation was based on the new cases of absenteeism due to MSD each year/people at risk of becoming ill, excluding in the denominator individuals who were absent due to MSD in the years prior to the cohort period. For the calculation of the cumulative incidence of absenteeism due to MSD over the entire study period, all new cases that emerged during this period were considered (first sick leave due to MSD/population at the beginning of the period).

With regard to the sick leaves presented by civil servants, frequency and duration indicators were calculated, and the diagnoses included in sick leave certificates were described. The more frequent diagnoses were described, identified by the number of episodes of sick leave, and average duration of sick leaves were calculated for each diagnosis through the absenteeism duration index (ADI = total number of days of sick leave/number of episodes of sick leave).

In the analytic stage, the variable response was defined as the time up to the first sick leave due to MSD. Censored cases were those that, over the observation period - from the study baseline date up to study completion date (December 31, 2016) -, did not take a sick leave due to MSD, or those that were excluded from the cohort due to death, transfer to another agency, dismissal, or retirement.

Predictor variables were defined on the follow-up start date. Sociodemographic variables included sex and age, whereas occupational variables included time of service, position, and area of work. Observation time was individually calculated for each worker based on the number of days from the study baseline date up to observation end date, which was when the worker was excluded from the cohort or the date of the first sick leave due to MSD.

The Kaplan-Meier estimator was used to estimate survival functions and the Cox model to test the proportional hazard assumption. The identification of risk factors for sick leave due to MSD was based on hazard ratio (HR), by applying Cox regression. The variable time of service showed high correlation (0.69) with the variable “age”, and was thus excluded from the model. All the other independent variables were maintained in the final regression model.

Considering that the entire target population of the study was included, no probability sampling plan was employed, and no statistical inference procedures were applied. Thus, neither p-values nor confidence intervals were reported.^8^ Data processing was conducted using an electronic spreadsheet. Analyses, graphs, and tables were created using the R software, version 3.3.1, and SPSS 20.

This research was approved by the Research Ethics Committee of Faculdade de Medicina da Bahia and conducted according to the standards provided in Resolution 466/12 and Operating Standard 001/13 of the National Health Council.

## RESULTS

At baseline, the study population consisted of 2,662 civil servants. A total of 614 participants were excluded due to different reasons (death, relocation to another agency, dismissal, and retirement). Men accounted for 47% of the study population, and women accounted for 53%. Nearly half (1,511) of workers were older than 40 years (56.8%), with a mean age of 42 years and a median age of 39 years, ranging from 21 to 57 years (Table 1).

**Table 1:** Sociodemographic and occupational characteristics of civil servants at a judiciary court in the state of Bahia, Brazil

Variables	n	%
Sex		
Male	1,253	47.1
Female	1,409	52.9
Age (years)		
Up to 30	435	16.3
> 30 up to 40	716	26.9
> 40 up to 50	990	37.2
> 50	521	19.6
Schooling		
< Higher education	448	16.8
Higher education	2,214	83.2
Position		
Analyst	819	30.8
Judge	215	8.1
Technician	1,628	61.2
Area of work		
Administrative	1,115	41.9
Judiciary	1,547	58.1
Time of service (years)		
Up to 10	907	34.1
> 10 up to 20	545	20.5
> 20 up to 30	990	37.2
> 30	220	8.3
Total	2,662	100

Individuals with higher education represented 83.2% (2.214) of the study population. However, this study revealed that, despite having a high educational level, most servants were judicial technicians, who accounted for 61.2% (1,628) of workers. Nearly 58% of servants (1,547) worked in the judiciary area, and 45.5% (1,210) had above 20 years of service (Table 1).

The greatest incidences of sick leaves due to MSD were reported in 2011 (6.9) and 2012 (6.0), which corresponded to the first years of follow-up. It bears noting that, throughout the study period, there was a higher annual incidence of absenteeism due to MSD among women (6.5 to 4.5), individuals aged > 40 years (9.7 to 4.0), with no higher education (12.7 to 5.9), who held technical positions (8.7 to 3.6), who worked in the administrative area (8.3 to 4.4), and those with more than 30 years of service (11.4 to 5.0) (Table 2).

**Table 2:** Annual cumulative incidence of absenteeism due to musculoskeletal diseases (per 100 workers) according to sociodemographic and occupational variables in civil servants at a judiciary court in the state of Bahia, Brazil, 2011 to 2016

Variable	2011	2012	2013	2014	2015	2016	Mean annual incidence
Sex							
Male	7.3	4.4	4.0	3.1	2.5	2.3	3.9
Female	6.5	7.4	4.8	4.4	3.3	4.5	5.1
Age							
Up to 30	1.8	3.5	3.1	1.6	1.8	1.6	2.2
> 30 to 40	4.1	2.8	3.8	2.9	3.1	3.5	3.3
> 40	9.7	8.3	5.1	4.8	3.2	4.0	5.8
Schooling							
< Higher education	12.7	10.3	5.8	3.4	3.8	5.9	7.0
Higher education	5.7	5.2	4.2	3.8	2.8	3.1	4.1
Position							
Analyst	3.9	4.6	4.2	3.5	2.6	3.0	3.6
Judge	4.2	6.2	2.9	1.2	0.6	3.9	3.2
Technician	8.7	6.7	4.8	4.3	3.5	3.6	5.3
Area							
Administrative	8.3	7.0	4.5	5.0	3.0	4.4	5.4
Judiciary	5.8	5.3	4.4	3.1	2.9	3.0	4.1
Time of service (years)							
Up to 10	3.3	4.2	3.9	3.3	2.4	2.0	3.2
> 10 to 20	7.2	5.4	3.8	3.8	2.0	2.6	4.1
> 20 to 30	9.0	7.0	5.0	3.6	3.2	4.6	5.4
> 30	11.4	9.9	5.4	6.1	6.1	5.0	7.3
Total	6.9	6.0	4.4	3.7	2.9	3.4	4.6

Table 3 shows that, during the follow-up period, 594 workers had at least one episode of sick leave due to MSD, with a cumulative incidence of 22%. Among the workers who were absent due to MSD over the 6 years (594), 254 (42.8%) had one episode of sick leave, and 174 (29.3%) had two or three episodes. It was observed that 53 workers (8.9%) took 10 or more sick leaves over the 6 years. Of the 356 (59.9%) workers whose sick leave lasted up to 15 days, 236 had only one episode of sick leave in the period, totaling 93% of short-term sick leaves. Conversely, among the 238 (40.1%) whose sick leaves lasted for more than 15 days, 220 had two or more episodes, accounting for 92.5% of long-term sick leaves.

**Table 3:** Distribution of episodes of absenteeism due to musculoskeletal diseases according to duration and frequency of sick leaves among civil servants at a judiciary court in the state of Bahia, Brazil, 2011 to 2016

Number of episodes	Duration of absence (days)				Total	
	≤ 15 days		> 15 days			
	n	%	n	%	n	%
1	236	92.9	18	7.1	254	100.0
2 to 3	99	56.9	75	43.1	174	100.0
4 to 9	21	18.6	92	81.4	113	100.0
10 or more	0	0.0	53	100.0	53	100.0
Total	356	59.9	238	40.1	594	100.0

There was a greater frequency of sick leaves due to MSD (n = 2,123, 19.6%), followed by respiratory tract diseases (n = 1,206, 11.2%), and mental and behavioral disorders (n = 1,084, 10.1%).With regard to sick leave duration, MSDs accounted for 14 days/episode, on average, which was lower than the average duration for other disease groups, such as mental disorders (27 days) and neoplasms (24 days) (data not shown).

The stratification of sick leaves due to MSD by ICD-10 revealed that dorsopathies (M40-54) were the most frequent diseases (51.5% of sick leaves), with back pain (M54) being the leading cause of sick leave in this category. Among soft tissue disorders (M60-79), the main diagnosis was shoulder lesions (M75), followed by synovitis and tenosynovitis (Table 4).

**Table 4:** Frequency and duration of episodes of absenteeism due to musculoskeletal diseases (MSD) according to International Classification of Diseases (ICD-10) subgroups among civil servants at a judiciary court in the state of Bahia, Brazil, 2011 to 2016

ICD subgroup (Ordinance no. 1339/MS) - MSD	n	%	Days	%	ADI*
G13 - Systemic atrophies primarily affecting central nervous system	1	0.05	30	0.10	30.0
G50-G59 - Nerve, nerve root and plexus disorders	100	4.71	2,521	8.47	25.2
G60-G64 - Polyneuropathies and other disorders of the peripheral nervous system	2	0.09	11	0.04	5.5
M00-M25 - Arthropaties	248	11.68	2,958	9.93	11.9
M40-M54 - Dorsopathies	1,093	51.48	10,173	34.16	9.3
M60-M79 - Soft tissue disorders	679	31.98	14,084	47.30	20.7
Total	2,123	100.00	29,777	100.00	14.0

* ADI = total number of days of sick leave/number of episodes of sick leave.

With regard to mean sick leave duration (ADI), soft tissue disorders stood out, especially those recorded as being related to excessive use (40 days), to tendon spontaneous rupture (35 days) and to shoulder lesions (27 days). Mononeuropathies of upper limbs also stood out, with a mean sick leave duration of 26 days (Table 4).

At the end of the study, it was found that workers of the judiciary sector had a likelihood of sickness absence due to MSD of 23%. Through the overall expression of the Kaplan-Meier estimator, considering the intervals in years and all healthy workers at *t* = 0 (January 1, 2011), it was observed that the likelihood of survival related to sick leave due to MSD throughout the 2,190 days (6 years) was estimated at 77%; therefore, 2,068 workers were censored at the end of the study.

In comparison to men, female workers had 39% greater rate of sick leave due to MSD (HR 1.39). Individuals older than 40 years had a 157% greater likelihood of absenteeism than young workers (HR 2.57). Those working in technical positions had a 48% greater risk compared to judges/analysts (HR 1.48). Professionals working in the administrative area had a 30% greater likelihood of sick leave due to MSD (HR 1.30) (Table 5).

**Table 5:** Final Cox regression model with risk factors for absenteeism due to MSD in civil servants at a judiciary court in the state of Bahia, Brazil, 2011 to 2016 (n = 2,662)

Variables	Sick leave due to MSD (yes:no)	Unadjusted HR	Adjusted HR
Sex			
Male	247:1,253	1	1
Female	347:1,409	1.20	1.39
Age (years)			
< 30	47:435	1	1
30 to 40	119:716	1.46	1.43
> 40	428:1,511	2.27	2.57
Position			
Judge/analyst	184:1,034	1	1
Technician	410:1,628	1.33	1.45
Area			
Judiciary	324:1,547	1	1
Administrative	270:1,115	1.12	1.30

HR = hazard ratio; MSD = musculoskeletal diseases.

## DISCUSSION

In this study, absenteeism due to MSD was identified through sick leave records, i.e., medical diagnosis of diseases affecting the musculoskeletal system. Therefore, the frequency of absenteeism due to MSD, as expected, was lower than that observed in findings from epidemiological studies that addressed complaints of musculoskeletal pain instead of disability, which is the object of the present study. In a study with workers from urban cleaning services, 30% reported multisite pain within the 7 days prior to investigation,^1^ and 57% in the last 12 months.^9^ This prevalence of multisite pain was strongly associated with health care utilization and work disability, which determined absenteeism or restricted work.^9^ These findings point to high morbidity rates for MSDs and a significant impact on work disabilities.

In the present study, the incidence of absenteeism due to MSD ranged from 7.3 to 2.3% in men and from 6.5 to 4.5% in women over the years of follow-up. It bears highlighting that women presented higher incidences in most observed years, with the greatest mean annual incidence of absenteeism due to MSD. Furthermore, individuals with schooling lower than higher education showed annual incidences of MSD greater than those of individuals with higher education throughout the entire period. These data are similar to those of a study with Finish workers in which incident cases of absenteeism due to MSD ranged from 6 to 5% in women and from 4.6% to 3.6% in men.^10^

Similar results were observed in a prospective study on absenteeism with Norwegian workers,^11^ showing greater incidences in women, older adults, and less educated people. In contrast, a follow-up study with data from Danish workers^12^ did not reveal a significant association of sex and age with absenteeism due to MSD and found that this condition was associated only with physical exertion, manual work, low social support, smoking, and multisite pain, variables that were not the object of the present study. Other studies highlight some factors that have been pointed out as promoting factors of sick leave due to MSD in women, such as working in positions requiring greater manual dexterity and accuracy; excessive working hours (double shift); authoritarian management towards women, with historical and cultural roots, in the work environment; less muscle strength compared with men; and exposure to physical and social stressors.^13,14^

With regard to the incidence of absenteeism among older adults, it has been discussed that increased age may have an impact on reduced capacity of tissue recovery and on the consequent accumulated tensions,^15^ leading to an increased incidence of MSD and thus of absenteeism. A study with Finish workers revealed a high incidence of permanent absenteeism due to MSD in older populations.^15^ This finding reveals the need to promote interventions in this group vulnerable to permanent sick leave. Another study on absenteeism due to MSD with 47 occupational groups in 18 countries revealed an association between advanced age, low schooling, and physical loading at work.^16^

Although most of workers of the study population had higher education, they worked in a technical position. Lower schooling may be a determining factor for labor market insertion through more physically demanding jobs. A study with Finish workers observed that less educated individuals, who supposedly perform technical roles, show a greater frequency of long-term sick leave, compared to workers who perform supervision activities.^17^ A study with Austrian workers highlighted the greater association of absenteeism due to musculoskeletal pain and low schooling, low occupational levels, and low income.^18^

During the study period, there was a higher frequency of short-term sick leaves (15 days or less) and with only one absence episode. Noteworthy, the frequency of sick leaves was only one episode in 43% of sick leaves due to MSD, with 60% of workers being absent for less than 15 days. These findings are consistent with those of a study conducted in Finland, which also revealed that long-term sick leaves account for a small portion of the overall number of episodes of absenteeism and disproportionally contribute to overall costs of absenteeism.^19^

Among the diagnosis of sick leaves in population working in the judiciary sector, MSDs were the most frequent ones, accounting for nearly 20% of all sick leaves in the 6 years of follow-up (n = 2.123) and for the greater number of absents days, totaling 25% (n = 29,777) of the days of sick leaves for all causes. A similar finding was observed in an oil company, where there was a greater proportion of episode of absenteeism due to MSD.^20^ In studies on absenteeism in the public service, there was a greater frequency of sick leave due to MSD among the civil servants of the São Paulo State Health Department in the city of São Paulo, Brazil,^21^ of the Santa Catarina State Department of Administration, Brazil,^22^ and among workers from a public bank in the state of Minas Gerais, Brazil.^23^ A study with workers from a Brazilian federal university also observed higher rates of sick leave due to MSD and found that neoplasms and infectious diseases were associated with long-term sick leaves.^24^ Therefore, MSDs stood out by their high frequency of absenteeism in the public service, and servants in the judiciary sector, exposed to physical and mental stressors, are among these workers.

The greatest frequency of absenteeism due to MSD in this study was related to dorsopathies (ICD-10/M40-54), with a frequency of 51.5%, followed by soft tissue disorders (M60-79), with 32%. Among the dorsopathies, back pain, or low back pain (38.5%) stand out; among soft tissue disorders, shoulder lesions (11.7%), and synovitis and tenosynovitis (8.8%) stand out. With regard to mean sick leave duration, shoulder lesions resulted in 27 days of sick leave, followed by mononeuropathies of upper limbs (26 days). Although very frequent, dorsopathies resulted in shorter sick leaves, with a mean of 9.3 days. These findings were similar to those of other studies in terms of reasons for sick leave.^9,13,14,20,25^

The literature highlights that heavy physical work, awkward biomechanical postures in trunk flexion and rotation, upper limb elevation, and excessive repetition, associated with time demands of production, organization of ways of working, and inappropriate physical structure, increase the occurrence of MSD in the spine and in upper limbs.^5^ Workers participating in the present study developed roles related to the administrative and judiciary areas in different sectors, with demands ranging from public attendance services and organization of jurisprudence to procedural trials, requiring static positions of the spine, in predominantly seated work, with muscle activity centered on upper limbs.

From the perspective of biomechanical demands on the body, an extensive literature is dedicated to diseases that compromise the spine, with special emphasis on back pain and low back pain, which, although having great importance among workers who perform operational activities involving load handling, are also relevant in administrative workers who predominantly remain seated for long periods.^9^ Shoulder lesions are also one of the most prevalent MSDs, both when occurring as the only site of pain in the body and, especially, when occurring in association with pain in the neck and in upper back.^1^ This joint is particularly demanded, either in dynamic or static activities, leading to postural overload on the pectoral girdle.

MSDs significantly contributed to the overall number of sick leave days. A study with workers in many different occupations that used data from the Information System of Notifiable Diseases revealed that there were 5 million of working days lost, for 18,611 absent workers notified with MSD.^26^ The groups that stood out in the analysis were the illiterate people, in the age group from 50 to 59 years, with daily working hours higher than 6 hours, with vertebral and cervical disc disorders, and with mental disorders.^26^ This findings evidenced the impact of MSDs on the overall burden of absenteeism in this population. As the main recommendation, results point out to the adoption of measures to reduce MSDs and incapacity to work caused by this morbidity.

Being a female, being aged older than 40 years, occupying the position of judiciary technician, and working in the administrative area were predictors of absenteeism due to MSD among the employees at a judiciary court analyzed in this study. These findings corroborate the results of an investigation with Danish workers, in which low socioeconomic status, female sex, and multisite pain were associated with long-term sick leaves.^16^

In an investigation with female employees in the eldercare sector in Denmark, an association was observed between long-term sick leave and sedentary lifestyle, high body mass index, and smoking.^27^ The explanation for these characteristics has a strong pathophysiological component, i.e., the role of physical inactivity, overweight, and smoking for the occurrence of MSDs has been well demonstrated in the literature, especially in longitudinal studies.^5^ Therefore, the finding of the present study that the rate of sick leaves due to MSD is almost 40% greater among women is consistent with the literature, and may also be explained by the characteristics pointed out by the aforementioned Danish study. However, our retrospective cohort did not have data on physical activity practice, level of physical inactivity, anthropometric levels, and smoking status, which would have allowed to analyze the role of these factors in the development of MSD. This is a common limitation in studies with secondary data based on medical records related to sick leaves.

With regard to the national literature, a study with workers in the municipality of Goiânia, Brazil, identified female sex, age older than 45 years, and low schooling as predictors of sick leave due to MSD,^28^ findings similar to those of the present study. More recently, studies on MSD have addressed concurrent, multisite pain^1,9^ and its role in incapacity to work and absenteeism. Among Finish workers, after 6 years of follow-up, it was observed that the number of painful sites is an important predictor of absenteeism due to MSD.^16^ Corroborating these findings, a study conducted in a food industry company revealed that, regardless of physical exposure at work, multisite pain is a predictor of absenteeism due to MSD.^29^ Furthermore, a Swedish study found an association between neck, shoulder, and low back musculoskeletal disorders and short-term absenteeism.^30^

In the present study, disability due to MSD was analyzed with the clinical manifestation of pain in several body sites and with different diagnoses. Although servants may have presented other episodes after their first sick leave due to MSD, the analysis was limited to first episodes in the study period and did not include absenteeism trajectory. Therefore, these analytical perspectives of investigating concurrent pain and assessing absenteeism trajectory are promising lines of research on absenteeism and MSD in future studies with this population.

One of the strengths of this study is its longitudinal design, with a 6-year follow-up of the entire population of workers in the judiciary sector in the state of Bahia, including different occupations and workplaces. The investigation of civil servants, i.e., workers with job stability, minimizes losses that are common in follow-up, especially in private work environments. Data were derived from medical records that provide a specific diagnosis, reducing classification errors in the estimation of MSD incidence.

## CONCLUSIONS

Considering the major impact of MSDs on workers’ health, it is important to highlight the need of preventive actions aimed at reducing MSDs and occupational disability resulting from them, as well as promoting servants’ health. Women, older adults, and individuals who hold technical positions are worthy of attention from the health management department of the court, since they presented the highest rates of incapacity to work during the study period.

The study was conducted with secondary data, and researchers performed the analysis with the required methodological rigor, but the limited number of variables available in corporate databases did not allow for broadening explanations on the relationships between absenteeism due to MSD and occupational factors. However, the present research opens perspectives for future investigations on absenteeism due to MSD with concurrent or multisite pain. These findings should guide programs to reduce disability and preventing musculoskeletal disorders in the institution.
